# CD24 cell surface expression in Mvt1 mammary cancer cells serves as a biomarker for sensitivity to anti-IGF1R therapy

**DOI:** 10.1186/s13058-016-0711-7

**Published:** 2016-05-14

**Authors:** Ran Rostoker, Sarit Ben-Shmuel, Rola Rashed, Zila Shen Orr, Derek LeRoith

**Affiliations:** Diabetes and Metabolism Clinical Research Center of Excellence, Clinical Research Institute at Rambam (CRIR), Rambam Medical Center, Haifa, Israel; The Ruth and Bruce Rappaport Faculty of Medicine, Technion - Israel Institute of Technology, Haifa, Israel; Department of Medicine, Icahn School of Medicine at Mt Sinai, New York City, NY USA

**Keywords:** IGF1R, CD24, Mammary tumor

## Abstract

**Background:**

The pro-tumorigenic effects of the insulin-like growth factor receptor (IGF1R) are well described. IGF1R promotes cancer cell survival and proliferation and prevents apoptosis, and, additionally it was shown that IGF1R levels are significantly elevated in most common human malignancies including breast cancer. However, results from phase 3 clinical trials in unselected patients demonstrated lack of efficacy for anti-IGF1R therapy. These findings suggest that predictive biomarkers are greatly warranted in order to identify patients that will benefit from anti-IGF1R therapeutic strategies.

**Methods:**

Using the delivery of shRNA vectors into the Mvt1 cell line, we tested the role of the IGF1R in the development of mammary tumors. Based on CD24 cell surface expression, control and IGF1R-knockdown (IGF1R-KD) cells were FACS sorted into CD24^−^ and CD24^+^ subsets and further characterized in vitro. The tumorigenic capacity of each was determined following orthotopic inoculation into the mammary fat pad of female mice. Tumor cells were FACS characterized upon sacrifice to determine IGF1R effect on the plasticity of this cell’s phenotype. Metastatic capacity of the cells was assessed using the tail vein assay.

**Results:**

In this study we demonstrate that downregulation of the IGF1R specifically in cancer cells expressing CD24 on the cell surface membrane affect both their morphology (from mesenchymal-like into epithelial-like morphology) and phenotype in vitro. Moreover, we demonstrate that IGF1R-KD abolished both CD24^+^ cells capacity to form mammary tumors and lung metastatic lesions. We found in both cells and tumors a marked upregulation in CTFG and a significant reduction of SLP1 expression in the CD24^+^/IGF1R-KD; tumor-suppressor and tumor-promoting genes respectively.

Moreover, we demonstrate here that the IGF1R is essential for the maintenance of stem/progenitor-like cancer cells and we further demonstrate that IGF1R-KD induces in vivo differentiation of the CD24^+^ cells toward the CD24^-^ phenotype. This further supports the antitumorigenic effects of IGF1R-KD, as we recently published that these differentiated cells demonstrate significantly lower tumorigenic capacity compared with their CD24^+^ counterparts.

**Conclusions:**

Taken together these findings suggest that CD24 cell surface expression may serve as a valuable biomarker in order to identify mammary tumors that will positively respond to targeted IGF1R therapies.

**Electronic supplementary material:**

The online version of this article (doi:10.1186/s13058-016-0711-7) contains supplementary material, which is available to authorized users.

## Background

The insulin-like growth factor receptor (IGFIR) is a transmembrane receptor tyrosine kinase [1] that is primarily activated by its cognate ligands, IGF1 and IGF2, and can be activated by insulin albeit with much lower affinity [2]. Since the early 1980s, when interest in IGF1R’s role in breast cancer development began [3, 4], preclinical studies demonstrated that IGF1R is involved in cell transformation in addition to mediating tumor-promoting functions such as proliferation, antiapoptosis and cancer cell dissemination [5–7]. Case control and prospective studies further supported the role of the IGF1R axis in breast cancer patients [8]. This repertoire placed IGF1R as a promising target for cancer therapy. However, recent results from clinical trials indicated that IGF1R antagonists failed to fulfill the high expectation [9, 10]. It has now become clear that predictive biomarkers are warranted in order to identify patients that will benefit from anti-IGF1R therapy.

Recently, based on CD24 cell surface expression we were able to distinguish, sort and maintain two distinct subpopulations of the metastatic mammary carcinoma cell line, Mvt1 that overexpresses the c-Myc and VEGF oncogenes [11]. CD24 is an anchored cell surface glycoprotein, mainly associated with the progression of invasive tumors through P-selectin binding, which is expressed by activated endothelial cells and platelets [12, 13]. More recently, it was shown that intracellular CD24 promotes tumorigenesis by the distribution of the ARF-NPM interaction and p53 inactivation [14]. Furthermore, it was shown that CD24 can serve as an important indicator for poor prognosis for many of the most common malignancies including breast cancer [15, 16].

Our recently published results demonstrated that CD24^+^ cells displayed more spindle-like cells that resemble a mesenchymal phenotype compared to the rounded epithelial morphology of their CD24^−^ counterparts. Moreover, CD24^+^ cells displayed highly tumorigenic and metastatic properties in vivo. Implantation of CD24^+^ cells into the mammary fat pad of FVB/N female mice and the hyperinsulinemic MKR female mice resulted in more rapidly growing tumors. Furthermore, this CD24^+^ subset formed metastatic lesions at a higher rate from both primary tumors and following tail vein injection.

CD24 is widely used to isolate pure mammary epithelial cells and along with other cell surface markers it can further serve to isolate stem/progenitor cells. It was shown that Lin^-^CD24^+^CD49f murine mammary cells were able to generate in vivo functional mammary tissue [17, 18]. In accordance with these findings, we have demonstrated that CD24^+^ cells possess cancer stem/progenitor-like properties both in vitro and in vivo. These cells are driven to differentiate in vivo and partly account for intratumor heterogeneity [19]. Whereas CD24 can serve in certain cases as a marker for stemness, the IGF1R was found to play a crucial role in maintaining pluripotent properties of human embryonic stem cells [20]; the self-renewal property of cancer stem cells [21].

In this study, we determined whether the cell surface expression of CD24 may serve as a valuable biomarker for tumor sensitivity to anti-IGF1R therapy. To begin to elucidate this issue we compared the effects of the IGF1R-knockdown (KD) on CD24^-^ and CD24^+^ cells morphology and phenotype in vitro. Moreover, we compared the tumorigenic and metastatic capacity in vivo of these distinct subsets following IGF1R-KD. We further screened for transcripts that may illuminate on the discrepancy in the effects obtained between these subsets. Finally, we tested the feasibility of the results on the human mammary cancer MCF7 cell line.

We anticipate that this study may constitute the starting point for future attempts to clinically circumvent the negative effects of IGF1R overstimulation in breast cancer patients.

## Methods

### Cell culture

Mouse and human mammary cancer cell lines, Mvt-1, and MCF7 cell line have been previously described [22, 23]. Cells were cultured in Dulbecco’s modified Eagle’s medium (DMEM; Biological Industries, Beit Haemek, Israel) supplemented with 10 % fetal bovine serum (FBS) (Biological Industries) and antibiotics (penicillin:streptomycin; Biological Industries) at 37 °C in a humidified atmosphere consisting of 5 % CO2 and 95 % air.

### Knockdown of IGF1R and IR by lentiviral-based delivery of shRNA

Vectors (GIPZ) encoding the following microRNA-adapted short hairpin RNAs (shRNA) 5’-TGACTGTGAAATCTTCGGC-3’ (mouse/human IGF1R), 5’- TTAGTTCCATGATGACCAG-3’ (mouse IGF1R) packed in high-titer lentiviral particles were purchased from Open Biosystems (Huntsville, AL, USA). These vectors or a vector containing a scrambled shRNA sequence (control shRNA; Open Biosystems) were infected in the presence of 8 μg/ml polybrene (Sigma-Aldrich, Rehovot, Israel) into Mvt-1 or MCF7 cells, all three vectors contained a green fluorescent protein (GFP) marker and puromycin resistance gene. Stable knockdown of the IGF1R was achieved by selection with 2 μg/ml puromycin (Sigma-Aldrich).

### Protein extraction and Western blot analysis

Western blot and densitometric analysis was carried out for protein detection in cells and tumor tissues as previously described [24]. Antibodies toward IGF1R and β-actin were purchased from Cell Signaling Technology, Danvers, MA, USA, antibody toward SLPI was purchased from Thermo Fisher Scientific, Rockford, IL, USA, and the matched secondary antibody conjugated with horseradish peroxidase was purchased from Jackson Laboratories, Bar Harbor, ME, USA.

### Animals

Female MKR mice and control mice on an FVB/N background were used in this study. The MKR mice are transgenic mice with a dominant-negative insulin-like growth factor-I receptor specifically targeted to the skeletal muscle, with a resultant severe insulin resistance and hyperinsulinemia phenotype [25]. Mice were kept on a 12-hour light/dark cycle with access to standard mouse chow and fresh water ad libitum. Mice studies were performed according to the protocol approved by the Technion Animal Inspection Committee. The Technion holds a National Institutes of Health (NIH) animal approval license number A5026-01.

### Syngeneic orthotopic tumor models

CD24^-^ and CD24^+^ cells or knockdown cells were suspended in 100 μl phosphate-buffered saline (PBS) and then injected (5 × 10^4^ cells/mouse, fewer cells were injected for the serial dilution experiments) into the left inguinal mammary fat pad (number 4) of 8-week-old female MKR mice. Tumor volume was monitored once a week with calipers and the volume was calculated in mm^3^ by the formula: (width2 × length × 0.5). Following sacrifice, tumors were removed and weighed, then flash frozen in liquid nitrogen and kept at -80 for further analysis.

### Flow cytometry

Pacific Blue-conjugated anti-mouse CD24 or PerCP/Cy5.5-conjugated anti-human CD24 (Biolegend, San Diego, CA, USA) were used for cell surface staining of the cells. 7-amino actinomycin D (7-AAD, Biolegend) was used to gate live cells. Cells were stained at a concentration of 5 × 10^6^ cells/ml of fluorescence-activated cell sorting (FACS) buffer (PBS containing 0.1 % bovine serum albumin [BSA]) for 20 minutes on ice in the dark, after which, the cells were washed twice and resuspended in FACS buffer containing 7-AAD. Stained cells were analyzed using the CyAn ADP Instrument (Dako-Cytomation, Glostrup, Denmark) and the FlowJo 7.25 analysis software (Tree Star, Ashland, OR, USA). Flow cytometry-based cell sorting for CD24^-^ IGF1R-KD and CD24^+^ IGF1R-KD cells was performed using FACSAria (BD Biosciences, San Jose, CA, USA).

### Tumorspheres

CD24^-^ IGF1R-KD and CD24^+^ IGF1R-KD cell suspensions were prepared and plated in nonadherent conditions at 600 cells/cm2 in DMEM F12 HAM medium (Sigma-Aldrich, Rehovot, Israel) containing 20 ng/ml basic fibroblast growth factor (bFGF) (Sigma-Aldrich), 20 ng/ml epidermal growth factor (EGF) (Sigma-Aldrich), 4 μg/ml of heparin (Sigma-Aldrich) and B-27 supplement (1:50 dilution, GIBCO, Burlington, ON, Canada), and cultured at 37 degree with 5 % CO2. Tumorsphere-forming efficiency (TFE) (%) was calculated after 5 days as follows: (number of tumorspheres (>50 mm in diameter) per well/number of cells seeded per well)*100. In order to assess self-renewal, primary tumorspheres were centrifuged at 115 × g for 5 minutes; the pellet was resuspended in 300 μl of 0.5 % trypsin/0.2 % EDTA for 3 min at 37 °C. Tumorspheres were disaggregated into single cell suspension with the use of a 25 G needle and syringe, (trypsin was neutralized with medium containing serum). Cells were centrifuged at 580 × g for 5 min, the pellet was resuspended in ice-cold PBS, and single cell suspension was assured under a microscope. Single cells were plated at the same seeding density that was used in the primary generation. Following 5 days in culture tumorspheres (>50 mm in diameter) were measured.

### Quantitative PCR reaction for cDNA products

Quantitative PCR was performed using Absolute Blue SYBR-Green ROX mix (Thermo Fisher Scientific, ABgene, Epsom, UK). RNA was extracted from treated Mvt-1 cells with the Total RNA Purification Kit (NORGEN Biotek Corp, Thorold, Canada) according to manufacturer's instructions, followed by single-stranded cDNA synthesis using the Verso™ reverse transcriptase (Thermo Fisher Scientific, ABgene). The expression measurement of the designated genes was performed with the Rotor-GeneTM 6000 system (Corbett Research, Sydney, Australia) and its software, ver. 1.7. The relative gene copy number was normalized using B2M as independent internal control gene, and calculated by the 2^-(Ct_(n)_-Ct_(normalizer)_) method.

### Tumor dissociation into single cells

Breast tumors were minced with scalpels and transferred to gentleMACS™ dissociator C-tubes (Miltenyi Biotec, Bergisch Gladbach, Germany) containing 5 ml of DMEM medium (Biological Industries, Beit Haemek, Israel) supplemented with 10 % FBS (Biological Industries). C-tubes were then connected to the gentleMACS™ dissociator and tumor dissociation was performed according to the manufacturer’s instructions. Minced tumors were incubated in the C-tubes for 45 min with 300 unit/ml collagenase I (Sigma-Aldrich, Rehovot, Israel) and 2 mg/ml dispase II (Roche Diagnostics, Mannheim, Germany) at 37 °C in a humidified atmosphere consisting of 5 % CO2 and 95 % air. Following incubation a second spin on the gentleMACS™ dissociator was performed, and the cells were then filtered through a 40-μm falcon strainer (Becton Dickinson, Franklin Lakes, NJ, USA).

### Tail vein metastasis assay

A total of 10,000 cells from each subset were injected through the tail vein of WT mice to assess lung metastatic activity. Mice were sacrificed 28 days following injection, lungs were removed and fixed, macrometastases were counted under the light microscope.

### Statistical analysis

All data are presented as mean ± standard error of the mean (SEM). Independent *t* test and the Mann-Whitney test was used for statistical analysis of unmatched groups; the Wilcoxon signed-rank test was used for matched group comparison, with *P* values < 0.05 considered statistically significant.

## Results

### CD24^+^ cells demonstrate significantly higher levels of the IGF1R

**Fig. 1:**
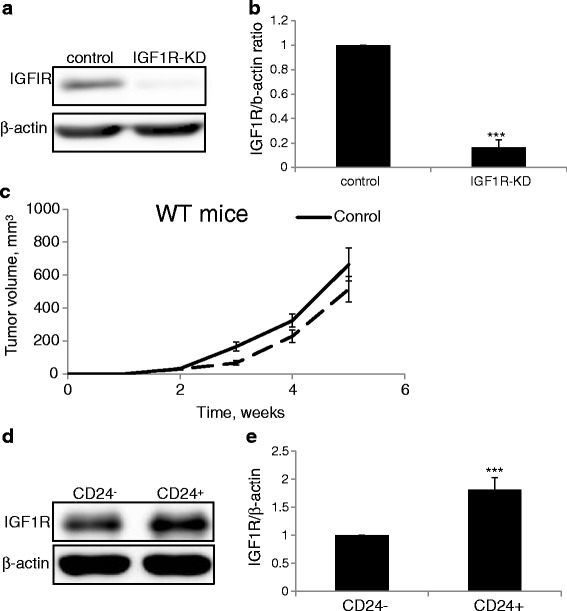
CD24^+^ cells demonstrate significantly higher levels of the IGF1R. **a** Western blot analysis of IGF1R expression in Mvt1 cells infected with control or IGF1R shRNA as indicated. **b** Protein expression was quantified relative to β-actin expression by densitometric analysis. **c** Control and IGF1R-KD cells were injected into the fourth mammary fat pad of 8-week-old virgin female FVB/N mice (50,000 cells/mouse) and tumor volume was measured during a 5-week period. **d** Western blot analysis of IGF1R expression in CD24^-^ and CD24^+^ Mvt1 cells. **e** Protein expression was quantified relative to β-actin expression by densitometric analysis. The Mann-Whitney test was performed to compare the difference in IGF1R between CD24^+^ and CD24^+^ cells ***P < 0.001. *IGF1R* insulin-like growth factor receptor, *KD* knockdown

In order to investigate the role of the IGF1R in tumorigenesis, we first downregulated the IGF1R in the Mvt1 cell line. IGF1R was downregulated by approximately 88 % as determined by Western blot analysis (Fig. 1a, b). Recently, we and others demonstrated that the efficacy of targeting IGF1R alone in cancer is limited [11, 26]. Here, we confirmed these results, as mammary tumors initiated by IGF1R-KD cells developed only slightly, but not significantly, slower compared to the control tumors in female FVB/N mice (Fig. 1c). CD24 expression serves as a marker for poor outcome in breast cancer patients [15], and we have recently demonstrated that CD24^+^ Mvt1 cells are highly tumorigenic compared with their CD24^-^ counterparts [19]. We therefore examined IGF1R levels in each of these subpopulations. Western blot analysis revealed significantly elevated IGF1R levels (>1.8-fold) in the aggressive CD24^+^ cells compared with the CD24^-^ subset (Fig. 1d, e).

### IGF1R-KD has a profound effect on CD24^+^ cells morphology and phenotype

**Fig. 2:**
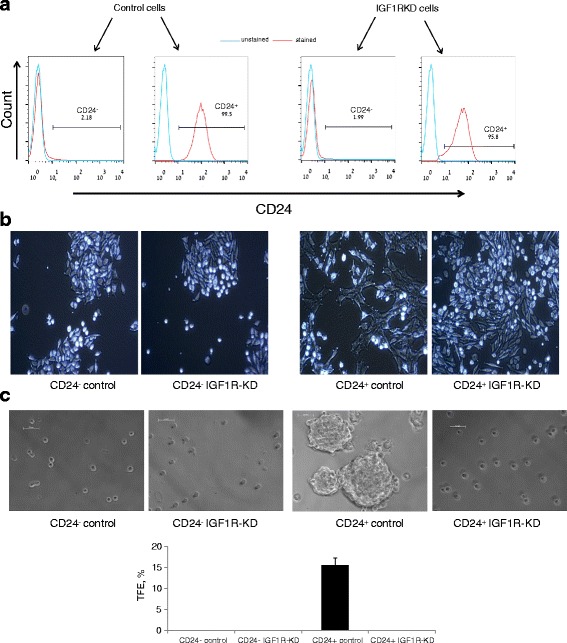
IGF1R-KD has a profound effect on CD24^+^ cells morphology and phenotype. **a** FACS histograms of control and IGF1R-KD Mvt-1 cells following sorting into pure CD24^-^ and CD24^+^ cell populations. **b** Cell phenotype in adherent culture for each group is presented in a phase-contrast bright field image. **c** Representative photomicrographs of tumorsphere (> 50 um diameter) grown from single cells in nonadherent culture for 5 days (*upper panel*). TFE (%) comparison between the groups (*lower panel*). *IGF1R* insulin-like growth factor receptor, *KD* knockdown, *TFE* tumorsphere formation efficiency

In order to test the effect of IGF1R-KD in each subset (CD24^-^ and CD24^+^ cells), control and IGF1R-KD cells were double sorted into pure (>95 % as determined by FACS analysis) CD24^-^ and CD24^+^ subpopulations (Fig. 2a). In accordance with our recent publication [19] CD24^+^ control cells displayed distinct morphology in adherent conditions compared to their CD24^-^ counterparts. CD24^+^ are larger cells with spindle-like cytoplasm compared to the more rounded-epithelial CD24^-^ control cells (Fig. 2b). Whereas IGF1R-KD had no effect on the CD24^-^ cell morphology, the CD24^+^ IGF1R-KD cells appeared to have a more epithelial morphology, similar to the CD24^-^ cells and distinct from the CD24^+^ control cells (Fig. 2b).

Using the tumorsphere assay we demonstrated that CD24^+^ Mvt-1 cells possess cancer stem-like cells characteristics [19], and we tested whether this phenotype is IGF1R-dependent. Cells were cultured in nonadherent conditions in serum-free optimized medium. As expected, CD24^+^ control cells displayed high tumor-forming efficiency. No tumorspheres were detected in the CD24^-^ control and CD24^-^ IGF1R-KD groups. In contrast, in the CD24^+^ cells, IGF1R-KD abolished the capacity to generate tumorspheres (Fig. 2c).

### Knockdown of the IGF1R in CD24^+^ Mvt1 cells impairs mammary tumor formation

**Fig. 3:**
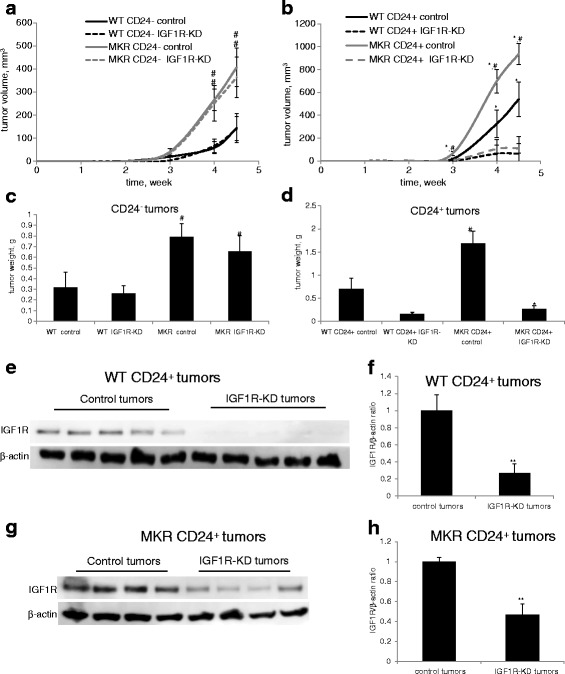
Knockdown of IGF1R in CD24^+^ Mvt1 cells impairs mammary tumor formation. Cells were injected into the mammary fat pad of 8-week-old WT and MKR female mice. Tumor volume (**a**, **b**) was monitored during 4.5 weeks and tumor weights (**c**, **d**) were measured at necropsy. Tumor lysates from WT (**e**) and MKR (**g**) mice were separated by SDS-PAGE, and IGF1R levels were assessed using Western blotting. **f**, **h** Relative expression was quantified by densitometric analysis and is presented as a fold change compared with the control group. Equal loading of proteins was demonstrated by immunoblotting with an antibody directed against β-actin. Mann-Whitney test was performed to compare the difference between the control tumors and IGF1R-KD tumors, *P < 0.05, and difference between WT and MKR tumors ^#^ *P* < 0.05. *IGF1R* insulin-like growth factor receptor, *KD* knockdown

Based on our in vitro results (Fig. 2b, c), we hypothesized that IGF1R-KD suppresses the tumorigenic capacity of the CD24^+^ Mvt1 cells. Hence, we compared tumor growth rate following inoculation of CD24^-^ control and CD24^-^ IGF1R-KD cells and following inoculation of CD24^+^ control and CD24^+^ IGF1R-KD cells into the mammary fat pad of WT and MKR female mice. In both WT and MKR mice, tumors developed with similar growth rates following inoculation of either CD24^-^ control or CD24^-^ IGF1R-KD cells, and as expected tumorgenicity was enhanced in hyperinsulinemic MKR mice compared with WT mice (Fig. 3a). On the other hand, inoculation of CD24^+^ IGF1R-KD cells resulted with significantly smaller tumors compared to the CD24^+^ control tumors in both WT and MKR mice (Fig. 3b). Tumor weights, at the termination of the experiment, confirmed these findings (Fig. 3c, d). These results were further confirmed with a different shRNA construct against the IGF1R (Additional file 1: Figure S1A-E). Next, tumors were lysed and subjected to Western blot analysis, which confirmed a significant reduction in IGF1R expression in the CD24^+^ IGF1R-KD tumors compared to the CD24^+^ control tumors (approximately 73 % and approximately 53 % in the WT mice (Fig. 3e, f) and MKR mice (Fig. 3g, h) respectively). It is important to note that pAkt levels and pERK levels were not different between the groups (data not shown). This may be attributed to several endogenous factors that can activate both the PI3K/Akt pathway and the MAPK pathway, or alternatively, these pathways are dynamic and the results observed are representative of a specific time point.

### SLPI is downregulated and CTGF is upregulated following IGF1R-KD in CD24^+^ mouse and human cells

**Fig. 4:**
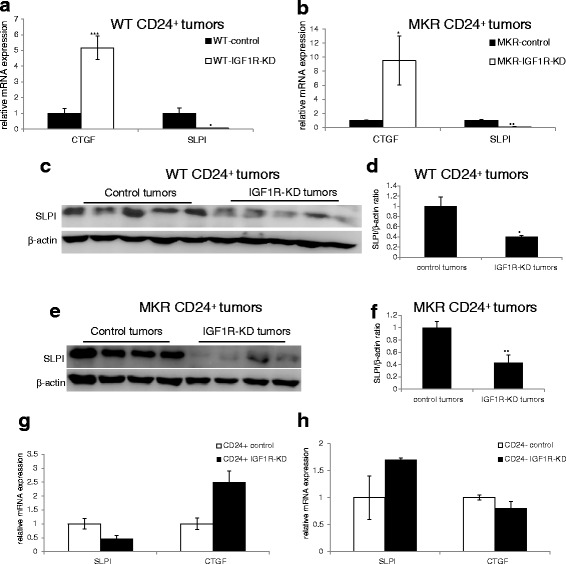
IGF1R-KD induces SLPI downregulation and CTGF upregulation in CD24^+^. SLPI and CTGF mRNA levels were determined by qRT-PCR analysis in WT (**a**) and MKR (**b**) tumors. Tumor lysates from WT (**c**) and MKR (**e**) mice were separated by SDS-PAGE, and SLPI levels were assessed using Western blotting. **d**, **f** Relative expression was quantified by densitometric analysis and is presented as a fold change compared with the control group. Equal loading of proteins was demonstrated by immunoblotting with an antibody directed against β-actin. **g**, **h** qRT-PCR analysis of SLPI and CTGF in vitro. Mann-Whitney test was performed to compare the difference between the control group and IGF1R-KD group, *P < 0.05, **P < 0.005, ***P < 0.001. *CTGF* connective tissue growth factor, *IGF1R* insulin-like growth factor receptor, *KD* knockdown, *mRNA* messenger RNA, *SLPI* secretory leukocyte protease inhibitor

In order to identify transcripts that may be elucidated for the specific effect of IGF1R on CD24^+^ tumorigenic capacity we screened by qtPCR analysis several transcripts that are associated with tumor growth and metastasis. Our results indicate significant changes in two transcripts with opposite effects on tumor growth. Connective tissue growth factor (CTGF) has been suggested as a tumor suppressor in human breast cancer [27] and the secretory leukocyte protease inhibitor (SLPI) that enhances tumor aggressiveness through vascular mimicry [28]. CTGF mRNA expression was significantly upregulated in the CD24^+^ IGF1R-KD tumors compared with the control tumors (>5-fold and >9-fold in the WT mice and MKR mice respectively (Fig. 4a, b)). On the other hand, SLPI expression was significantly down-regulated in the CD24^+^ IGF1R-KD tumors by >15 fold in the WT mice and by >9 fold in the MKR mice (Fig. 4a, b). SLPI protein levels were significantly reduced by approximately 60 % in the CD24+ IGF1R-KD tumors compared with the control tumors in both WT (Fig. 4c-d) and MKR mice (Fig. 4e-f).

In order to confirm that these effects are a direct result of IGF1R downregulation and are specific to the CD24^+^ cells, we compared the mRNA levels of these two transcripts in vitro. Our results, confirmed that the expression SLPI and CTGF is altered following IGF1R-KD. CD24^+^ IGF1R-KD cells displayed reduced SLPI levels (>2-fold) and elevated CTGF levels (2.5-fold) (Fig.4g) compared to the CD24^+^ control cells. These effects were specific to the CD24^+^ cells, since IGF1R-KD had no effect on either of these transcripts in the CD24^-^ cells (Fig. 4h). These results were further validated with the second construct against the IGF1R (Additional file 2: Figure S2). Additionally, we demonstrated that in the CD24^+^ MCF7 cell line IGF1R-KD results in an approximately 52 % reduction in SLPI mRNA expression as observed following IGF1R-KD in the Mvt-1 CD24^+^ cells (Additional file 3: Figure S3A, B).

### IGF1R-KD enhances CD24+ cancer cell plasticity in vivo

**Fig. 5:**
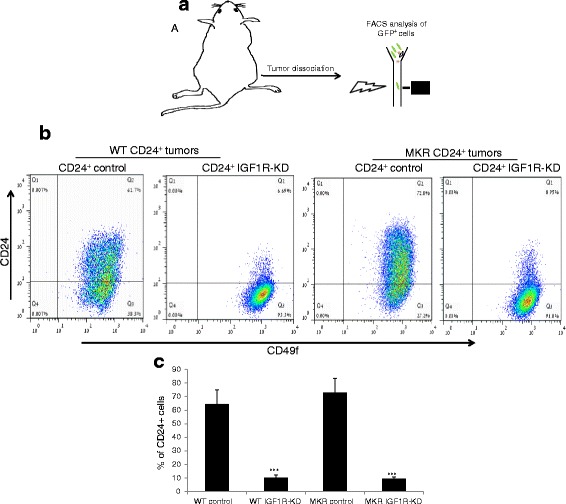
IGF1R-KD enhances CD24^+^ cancer cells plasticity in vivo. **a** Experimental strategy scheme is presented. CD24^+^ control or CD24^+^ IGF1R-KD cells were injected into the mammary fat pads, after 4 weeks, tumors were harvested and CD24 expression was evaluated. **b** CD24 expression in GFP-expressing cancer cells from mammary tumors was determined by FACS analysis. **c** Comparison of CD24^+^ population between control and IGF1R-KD tumors in WT and MKR mice. Mann-Whitney test was performed to compare between control and IGF1R-KD tumors, ^***^ *P* < 0.001. *FACS* fluorescence-activated cell sorting, *GFP* green fluorescent protein, *IGF1R* insulin-like growth factor receptor, *KD* knockdown

Recently, we have demonstrated a direct plasticity process whereby CD24^+^ cells differentiate in vivo into CD24^-^ cells [19]. Based on the in vitro results (Fig. 2c) we hypothesized that IGF1R is involved in this process. To examine this, CD24^+^ control and CD24^+^ IGF1R-KD cells were injected into the mammary fat pad of WT and MKR mice, and tumors were allowed to grow for five weeks. At sacrifice, tumors were dissociated into single cells and cancer cells were FACS analyzed (based on their GFP expression) for CD24 expression (Fig. 5a). As expected, direct plasticity toward the CD24^-^ phenotype was observed in both control and IGF1R-KD groups in WT and in the MKR mice following implantation of CD24^+^ cells (Fig. 5b). IGF1R-KD significantly enhanced this differentiation process, as shown by control tumors from WT mice that were comprised of approximately 64 % CD24^+^ cells whereas only approximately 10 % were found to be CD24^+^ following implantation of CD24^+^ IGF1R-KD cells (Fig. 5c). Similar results were shown in the MKR tumors, where approximately 73 % of the cancer cells were CD24^+^ following implantation of the CD24^+^ control cells, and only approximately 10 % were CD24^+^ in the CD24^+^ IGF1R-KD tumors (Fig. 5c).

We further demonstrate that this differentiation process toward the CD24^-^ phenotype in the CD24^+^/IGF1R-KD is significantly enhanced as a result of the tumor microenvironment; the distribution of CD24^-^ and CD24^+^ subsets was not affected in vitro as a result of the IGF1R-KD (Additional file 4: Figure S4).

### IGF1R-KD significantly reduced the metastatic capacity of CD24^+^ cells

**Fig. 6:**
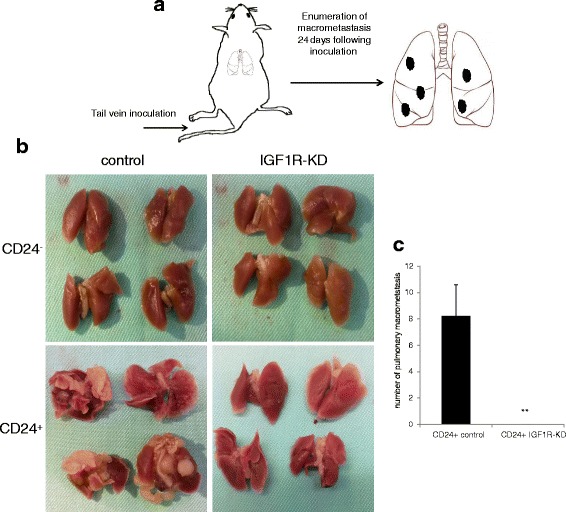
IGF1R-KD significantly reduced the metastatic capacity of CD24^+^ cells. **a** Experimental strategy scheme is presented. **b** Representation of lung metastasis following 4 weeks of 10,000 cells inoculation into the tail vein of the WT mice. **c** Average of macrometastasis per lung in each group is displayed in the bar graph. Mann-Whitney test performed to compare the difference between the groups. **P < 0.005. *IGF1R* insulin-like growth factor receptor, *KD* knockdown

We used the tail vein metastasis assay to determine the effect of IGF1R-KD in both subsets of cancer cells (Fig. 6a). Inoculation of each cell subset into the tail vein of WT mice revealed high metastatic capacity for the CD24^+^ control cells with approximately eight lesions per lung, whereas no lesions were found following CD24^-^ control cells inoculation (Fig. 6a, b). IGF1R-KD abolished CD24^+^ metastatic capacity, since no lesions were found following inoculation of the CD24^+^ IGF1R-KD cells. These results were further confirmed with the second construct against the IGF1R (Additional file 5: Figure S5).

## Discussion

Over the past few decades, findings regarding the role of the IGF1R in cancer have continued to accumulate. The IGF1R promotes cancer cell proliferation and survival and, on the other hand, prevents apoptosis [1, 29, 30]. Moreover, the importance of the IGF1R in establishing and maintaining a transformed phenotype was well described [31, 32]. These evidences positioned the IGF1R as a promising target for treating cancer. Hence, investigators along with pharmaceutical companies invested great efforts in developing therapeutic strategies toward the IGF system and specifically toward the IGF1R. Despite the great success in preclinical studies, results from recent and ongoing clinical trials have been distinctly disappointing [10]. It has become clear that predictive biomarkers are required in order to select patients who would benefit from anti-IGF1R therapy. In this study, we suggest that CD24 cell surface expression in cancer cells may predict sensitivity to anti-IGF1R therapy. Our results demonstrate that IGF1R-KD specifically in CD24^+^ cancer cells alters their morphology and phenotype and, more importantly, markedly inhibited their tumorigenic capacity.

In the current study, we demonstrate that IGF1R-KD had minimal effect on mammary tumors that formed by inoculation of the heterogeneous Mvt1 cell line. Recently, we found that the Mvt1 cells are comprised of two subpopulations that differ by their CD24 cell surface expression, therefore termed CD24^-^ and CD24^+^ cells [19]. Furthermore, our recent results, demonstrated that the CD24^+^ subset is highly tumorigenic; these cells formed rapidly growing mammary tumors and metastatic lesions compared to their CD24^-^ counterparts. Moreover, these cells displayed early stem/progenitor properties in vitro and in vivo [19]. Thus, we tested whether CD24 cell surface expression could serve as a new marker to predict response to anti-IGF1R therapy. We found that IGF1R-KD induces a rounded epithelial morphology in the CD24^+^ cells that are normally characterized by mesenchymal-like morphology, whereas CD24^-^ subset was not affected by the IGF1R-KD and maintained their epithelial morphology despite the IGF1R-KD. The ability of cells to generate tumorspheres when growing in suspension is a common method to identify cells with stem/progenitor properties [33]. The greater capacity of CD24^+^ cells to form tumorspheres and the inability of CD24^-^ cells to do so was the most profound phenotype discrepancy between these two populations in vitro [19]. Here we show that IGF1R-KD completely abolished CD24^+^ cells capacity to form tumorspheres.

In the pursuit of tailor-made medicine, the identification of biomarkers that can imply who should receive therapy and what therapy is greatly warranted [34, 35] Following the disappointing results of anti-IGF1R phase 3 clinical trials in unselected patients, the search for predictive biomarkers that can identify suitable patients who can benefit from anti-IGF1R therapy is now under intensive investigation [10]. In order to further investigate whether CD24 cell surface expression could implicate tumor sensitivity to anti-IGF1R therapy, we inoculated CD24^-^ and CD24^+^ cells with and without IGF1R-KD into the mammary fat pad of WT and the hyperinsulinemic MKR female mice. In accordance with our in vitro results, IGF1R-KD cells had a dramatic effect specifically on the CD24^+^ tumors. CD24^+^ IGF1R-KD cells formed significantly smaller tumors compared with the control CD24^+^, cells whereas IGF1R-KD had no effect on CD24^-^ tumor growth. Western blot analysis confirmed significant reduction of the IGF1R in both CD24^-^ and CD24^+^ tumors compared to the control tumors. It is important to note that the PI3K/Akt pathway and MAPK pathway failed to show a reduction, this may be attributing to other endogenous factors which can activate both pathways, and alternatively, this can reflect the specific time point of sacrifice since this protein modification is dynamic.

SLPI is a protease inhibitor and mediates a broad array of activities that may not be related to its antiprotease functions [36], including several malignancy-promoting functions [37]. In the search for proteins that enhance tumor formation and metastasis, Wagenblast et al, identified SLPI as a driver of metastatic formation in a mouse model for breast cancer. Additionally, SLPI emerged as the most significantly gene in human breast cancer patients that had lung-metastatic relapses [28]. Using RNA-seq analysis, we have identified SLPI among the top three upregulated genes in the highly tumorigenic CD24^+^ Mvt1 cell subset [19], and suggests that SLPI may play a role in the more aggressive cancers derived from CD24^+^ cells. Taken together we hypothesized that IGF1R-KD in the CD24^+^ significantly attenuates the tumorigenic capacity by regulating SLPI levels. Our results, confirmed a significant reduction in SLPI expression specifically in the CD24^+^ IGF1R-KD tumors compared with CD24^+^ control tumors. However, SLPI levels were comparable between CD24^-^ control and CD24^-^ IGF1R-KD tumors. We screened several other candidates that may elucidate the specific antitumorigenic effect of IGF1R-KD in the CD24^+^ tumors. We identified a significant upregulation in CTGF, specifically in the CD24^+^ IGF1R-KD tumors. CTGF is a member of the CCN family of proteins that are involved in adhesion, apoptosis, extracellular matrix production, and growth arrest of multiple cell types; this is mostly attributed to their ability to interact and activate integrins [27, 38]. Though the role the CCN proteins play in cancer is controversial, analysis of 122 human breast tumors suggested CTGF as a tumor suppressor [27]. We further demonstrated that this effect is not restricted to the tumor process and that SLPI and CTGF expression is directly regulated by IGF1R. QRT-PCR analysis of CD24^+^ cells in vitro following IGF1R-KD demonstrated that IGF1R downregulation results with a profound reduction in SLPI levels and upregulation in CTGF levels. In accordance with our in vivo results IGF1R-KD had no effect on both SLPI and CTGF mRNA levels in the CD24^-^ subset. The altered expression observed for both SLPI and CTGF following IGF1R-KD were dramatically greater in vivo. SLPI levels were reduced by approximately 10-fold in the CD24^+^ IGF1R-KD tumors (compared with the CD24^+^ control tumors), whereas the in vitro results demonstrated only a 50 % reduction. These in vivo changes in SLPI levels matched the difference in SLPI levels between CD24^+^ and CD24^-^ cells [19]. Recently, we demonstrated that CD24^+^ cells differentiate in vivo in response to intratumor stimuli into distinct CD24^-^ cell population [19]. This, along with recent reports that suggest a role for the IGF1R in maintaining stemness [20], encouraged us to test whether IGF1R-KD induced rapid differentiation in vivo toward the CD24^-^ phenotype. FACS analysis of cancer cells dissociated from CD24^+^ control and IGF1R-KD tumors, revealed that IGF1R is essential for the maintenance of stem/progenitors-like cancer cells that fuel the cancer process as recently described [19, 39].

There is some “heterogeneity” in the results of preclinical and clinical studies regarding anti-IGF1R therapies. Burtrum et al, demonstrated that IGF1R inhibition in vivo, resulted in a significant reduction in tumor growth following inoculation of the adenocarcinoma MCF7 cell line [5]. In contrast, primary tumors formed by MDA-MB-231 cells with IGF1R-KD grew as rapidly as the control tumors [40]. It is important to note that these cells known as CD24^-^ cells [41]. Our results indicate that CD24 expression in breast tumors may indicate sensitivity to anti-IGF1R therapy. Hence, to further investigate this, we determined CD24 cell surface expression in the mammary carcinoma MCF7 cell line. We found in accordance with previous studies [41, 42] that MCF7 are CD24^+^ cells. To test the feasibility of our results in CD24^+^ human breast cancer cells, we downregulated IGF1R in the MCF7 cells and identified a marked reduction in SLPI levels.

Dissemination of tumors cells from the primary tumors and colonialization in distant sites, accounts for the vast majority of cancer-related death [43]. Using the tail vein metastasis assay we demonstrated that IGF1R-KD abolished the metastatic capacity of the Mvt1 CD24^+^ cells.

## Conclusions

Taken all together, our results here indicate that cell surface expression of CD24 in breast tumor cells may serve as a valuable biomarker to identify breast cancer patients that will benefit from anti-IGF1R therapy. Moreover, we demonstrate that IGF1R play a significant role in maintaining cancer stem/progenitor-like phenotype of cancer cells, and these results in rapid tumor growth. Moreover, we identified that IGF1R regulates SLPI and CTGF expression specifically in CD24^+^ cells. Further research with different types of breast cancer models and different anti-IGF1R strategies are required in order to further determine the feasibility of these results.

## Additional files

Additional file 1: Figure S1. IGF1R-KD in CD24^+^ Mvt1 cells impairs mammary tumor formation. **(A)** Western blot analysis of IGF1R expression in Mvt1 cells infected with control or IGF1R shRNA as indicated. **(B)** Protein expression was quantified relative to β-actin expression by densitometric analysis. **(C)** Control and IGF1R-KD Mvt-1 cells were FACS sorted into pure CD24^-^ and CD24^+^ cell populations. **(D**) Control and IGF1R-KD cells were injected into the fourth mammary fat pad of 8-week-old virgin FVB/N mice (50,000 cells/mouse) and tumor volume was measured during a 5-week period **(E)** Tumor weights were measured at necropsy. Mann-Whitney test performed to compare the difference between the groups. **P* < 0. 05, ***P* < 0.005. (PPTX 107 kb) Additional file 2: Figure S2. IGF1R-KD induces SLPI downregulation and CTGF upregulation in CD24^+^. QRT-PCR analysis of SLPI and CTGF in vitro in Mvt1 cells. (PPTX 46 kb) Additional file 3: Figure S3. IGF1R-KD in the CD24^+^ MCF7 cell line, results in reduced SLPI expression. **(A)** FACS analysis of CD24 cell surface expression in MCF7 cells. **(B)** Western blot analysis of IGF1R expression in MCF7 cells infected with control or IGF1R shRNA as indicated. **(c)** QRT-PCR analysis of SLPI in vitro. (PPTX 1550 kb) Additional file 4: Figure S4 Cell surface expression of CD24 and CD49f. A FACS dot plot showing CD24 and CD49f cell surface expression in control and IGF1R-KD Mvt1 cells. (PPTX 72 kb) Additional file 5: Figure S5 IGF1R-KD significantly reduced the metastatic capacity of CD24^+^ cells. **(A)** Representation of lung metastasis following 4 weeks of 10,000 cells inoculation into WT mice tail vein. **(B)** Average of macrometastasis per lung in each group is displayed in the bar graph. Mann-Whitney test performed to compare the difference between the groups. ***P* < 0.005. (PPTX 537 kb)

## Abbreviations

BSA: bovine serum albumin
CTGF: connective tissue growth factor
DMEM: Dulbecco’s modified Eagle’s medium
FACS: fluorescence-activated cell sorting
FBS: fetal bovine serum
GFP: green fluorescent protein
IGF1R: insulin-like growth factor receptor
KD: knockdown
PBS: phosphate-buffered saline
SEM: standard error of the mean
shRNA: short hairpin RNA
SLPI: secretory leukocyte protease inhibitor
TFE: tumorsphere formation efficiency
