# Supplementary material for: CD24 cell surface expression in Mvt1 mammary cancer cells serves as a biomarker for sensitivity to anti-IGF1R therapy
Source: Breast Cancer Res. 2016 May 14;18:51. doi: 10.1186/s13058-016-0711-7 (PMC4867988; doi:10.1186/s13058-016-0711-7)
Supplement: Additional file 1: Figure S1. — IGF1R-KD in CD24+ Mvt1 cells impairs mammary tumor formation. (A) Western blot analysis of IGF1R expression in Mvt1 cells infected with control or IGF1R shRNA as indicated. (B) Protein expression was quantified relative to β-actin expression by densitometric analysis. (C) Control and IGF1R-KD Mvt-1 cells were FACS sorted into pure CD24- and CD24+ cell populations. (D) Control and IGF1R-KD cells were injected into the fourth mammary fat pad of 8-week-old virgin FVB/N mice (50,000 cells/mouse) and tumor volume was measured during a 5-week period (E) Tumor weights were measured at necropsy. Mann-Whitney test performed to compare the difference between the groups. *P < 0. 05, **P < 0.005. (PPTX 107 kb) [file 13058_2016_711_MOESM1_ESM.pptx]

## Slide 1
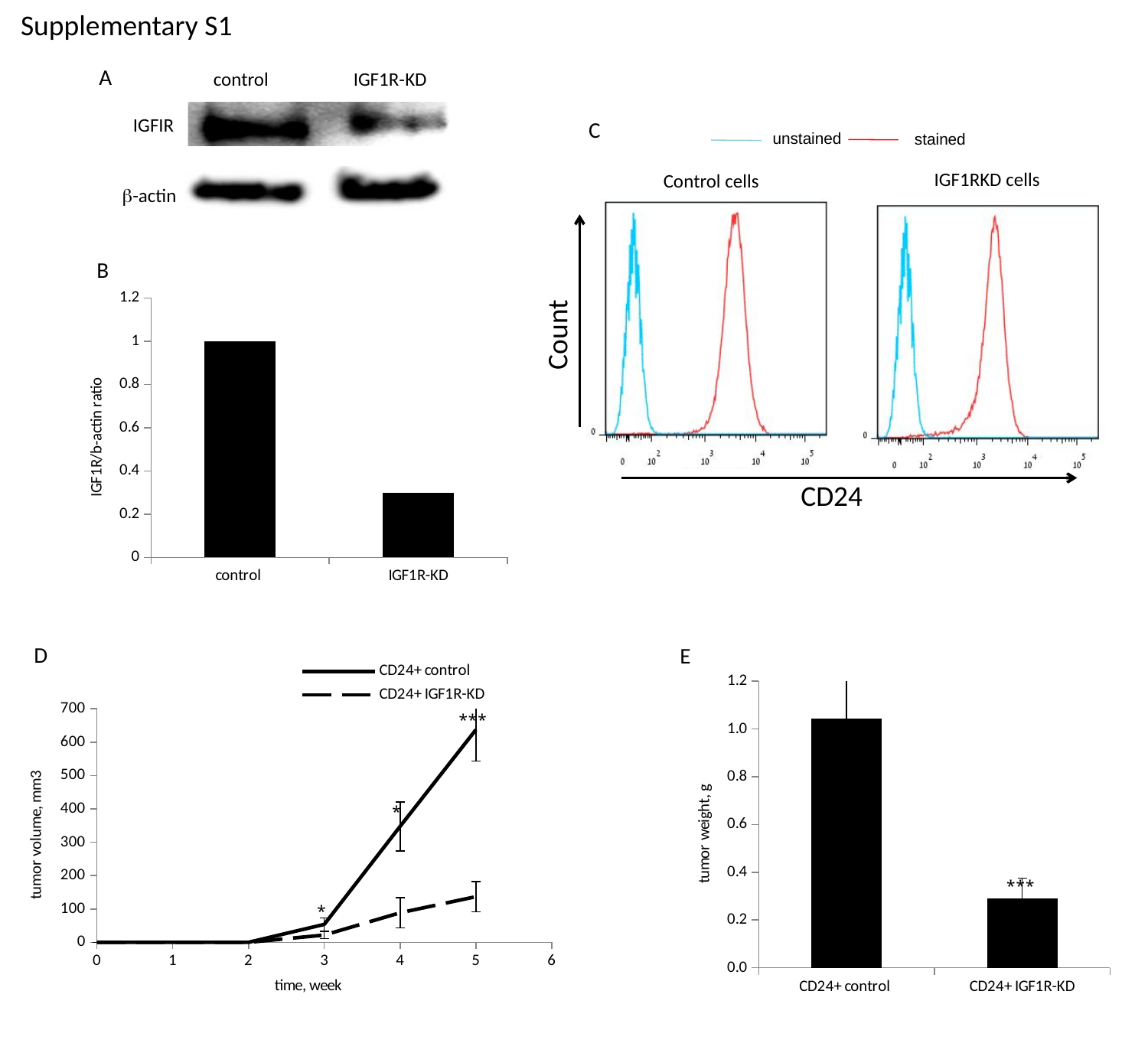

Supplementary S1
A
control
IGF1R-KD
IGFIR
b-actin
C
unstained
stained
IGF1RKD cells
Control cells
Count
CD24
B
### Chart
| Category | |
|---|---|
| control | 1.0 |
| IGF1R-KD | 0.3 |D
E
### Chart
| Category | CD24+ control | CD24+ IGF1R-KD |
|---|---|---|
### Chart
| Category | |
|---|---|
| CD24+ control | 1.044 |
| CD24+ IGF1R-KD | 0.291 |***
***
*
*
