# Supplementary figures and images for: CD24 cell surface expression in Mvt1 mammary cancer cells serves as a biomarker for sensitivity to anti-IGF1R therapy
Source: Breast Cancer Res. 2016 May 14;18:51. doi: 10.1186/s13058-016-0711-7 (PMC4867988; doi:10.1186/s13058-016-0711-7)

## Slide 1
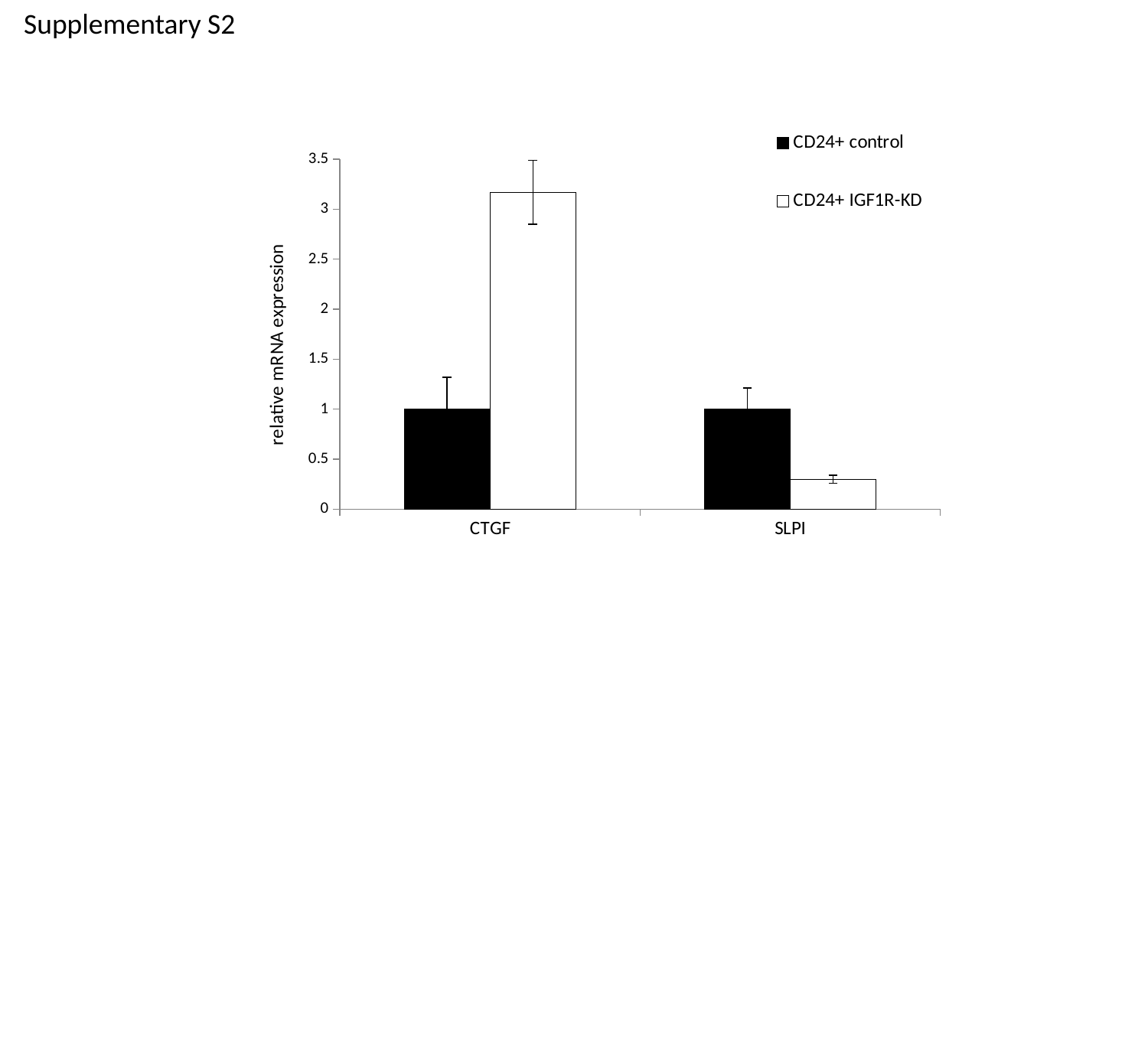

Supplementary S2
### Chart
| Category | CD24+ control | CD24+ IGF1R-KD |
|---|---|---|
| CTGF | 1.0 | 3.17 |
| SLPI | 1.0 | 0.3 |

Supplement: Additional file 2: Figure S2. — IGF1R-KD induces SLPI downregulation and CTGF upregulation in CD24+. QRT-PCR analysis of SLPI and CTGF in vitro in Mvt1 cells. (PPTX 46 kb) [file 13058_2016_711_MOESM2_ESM.pptx]

## Slide 1
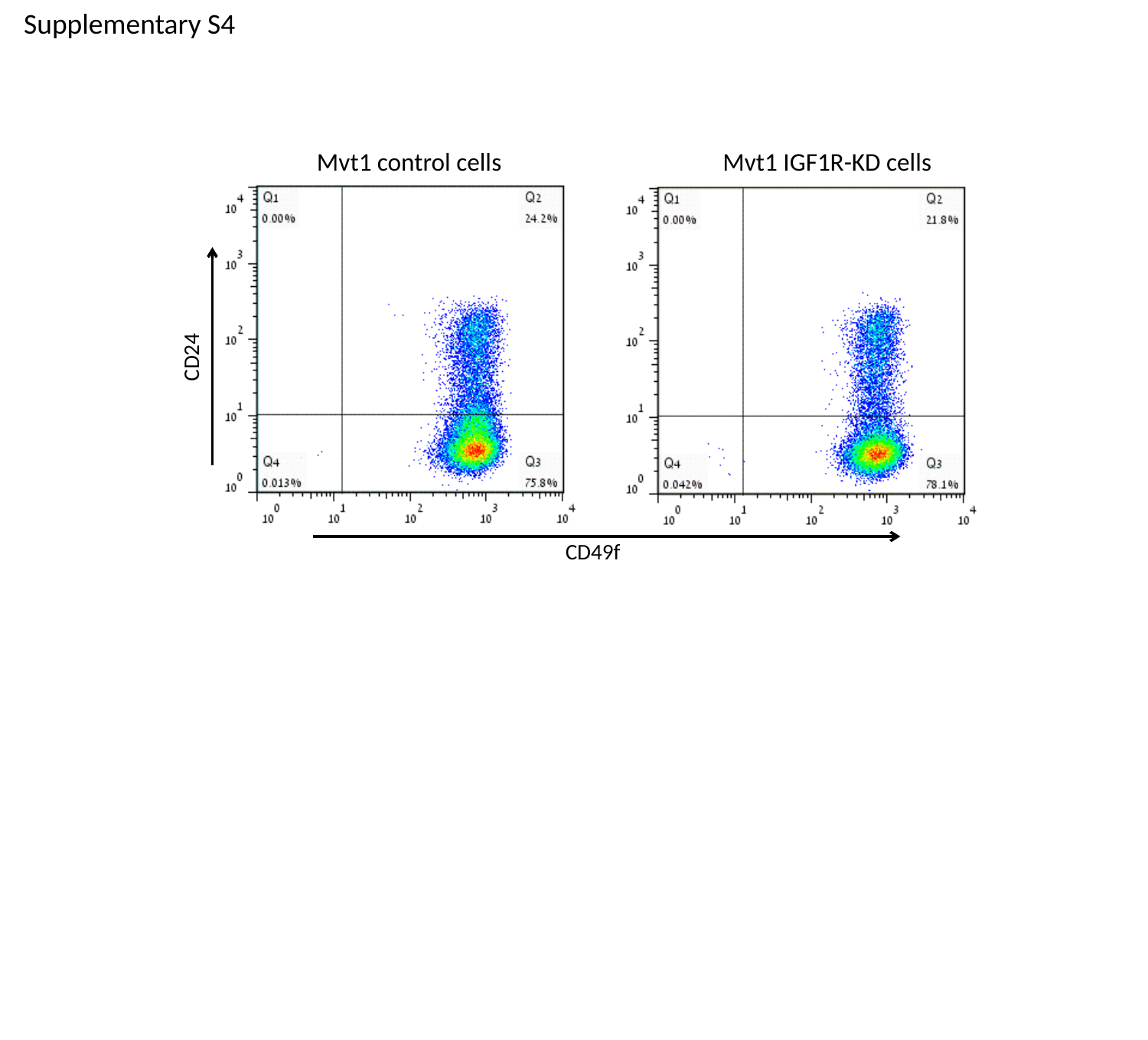

Supplementary S4
Mvt1 IGF1R-KD cells
Mvt1 control cells
CD24
CD49f

Supplement: Additional file 4: Figure S4 — Cell surface expression of CD24 and CD49f. A FACS dot plot showing CD24 and CD49f cell surface expression in control and IGF1R-KD Mvt1 cells. (PPTX 72 kb) [file 13058_2016_711_MOESM4_ESM.pptx]

## Slide 1
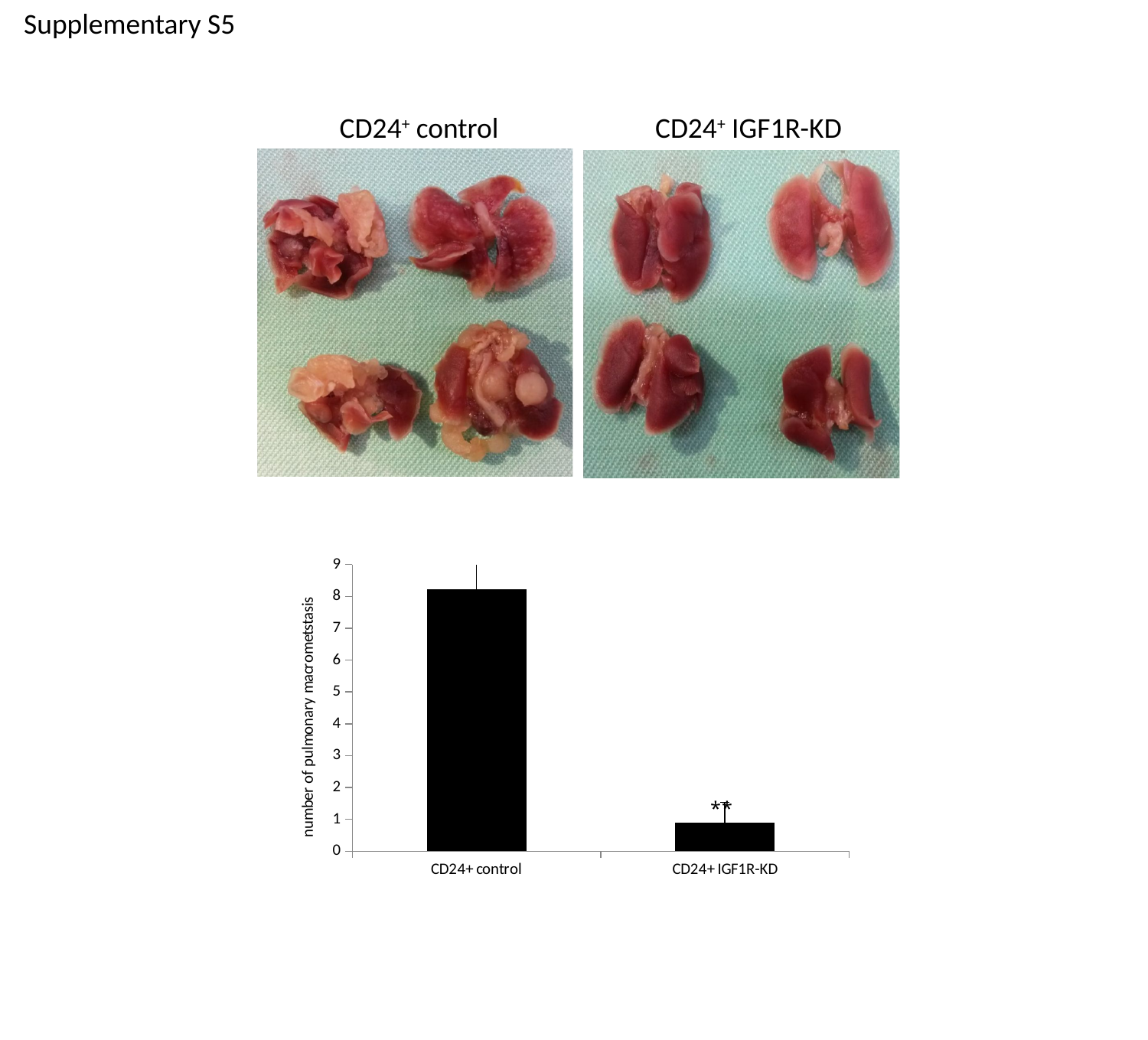

Supplementary S5
CD24+ control
CD24+ IGF1R-KD
### Chart
| Category | |
|---|---|
| CD24+ control | 8.222222222222221 |
| CD24+ IGF1R-KD | 0.9 |**

Supplement: Additional file 5: Figure S5 — IGF1R-KD significantly reduced the metastatic capacity of CD24+ cells. (A) Representation of lung metastasis following 4 weeks of 10,000 cells inoculation into WT mice tail vein. (B) Average of macrometastasis per lung in each group is displayed in the bar graph. Mann-Whitney test performed to compare the difference between the groups. **P < 0.005. (PPTX 537 kb) [file 13058_2016_711_MOESM5_ESM.pptx]
