# Supplementary material for: CD24 cell surface expression in Mvt1 mammary cancer cells serves as a biomarker for sensitivity to anti-IGF1R therapy
Source: Breast Cancer Res. 2016 May 14;18:51. doi: 10.1186/s13058-016-0711-7 (PMC4867988; doi:10.1186/s13058-016-0711-7)
Supplement: Additional file 3: Figure S3. — IGF1R-KD in the CD24+ MCF7 cell line, results in reduced SLPI expression. (A) FACS analysis of CD24 cell surface expression in MCF7 cells. (B) Western blot analysis of IGF1R expression in MCF7 cells infected with control or IGF1R shRNA as indicated. (c) QRT-PCR analysis of SLPI in vitro. (PPTX 1550 kb) [file 13058_2016_711_MOESM3_ESM.pptx]

## Slide 1
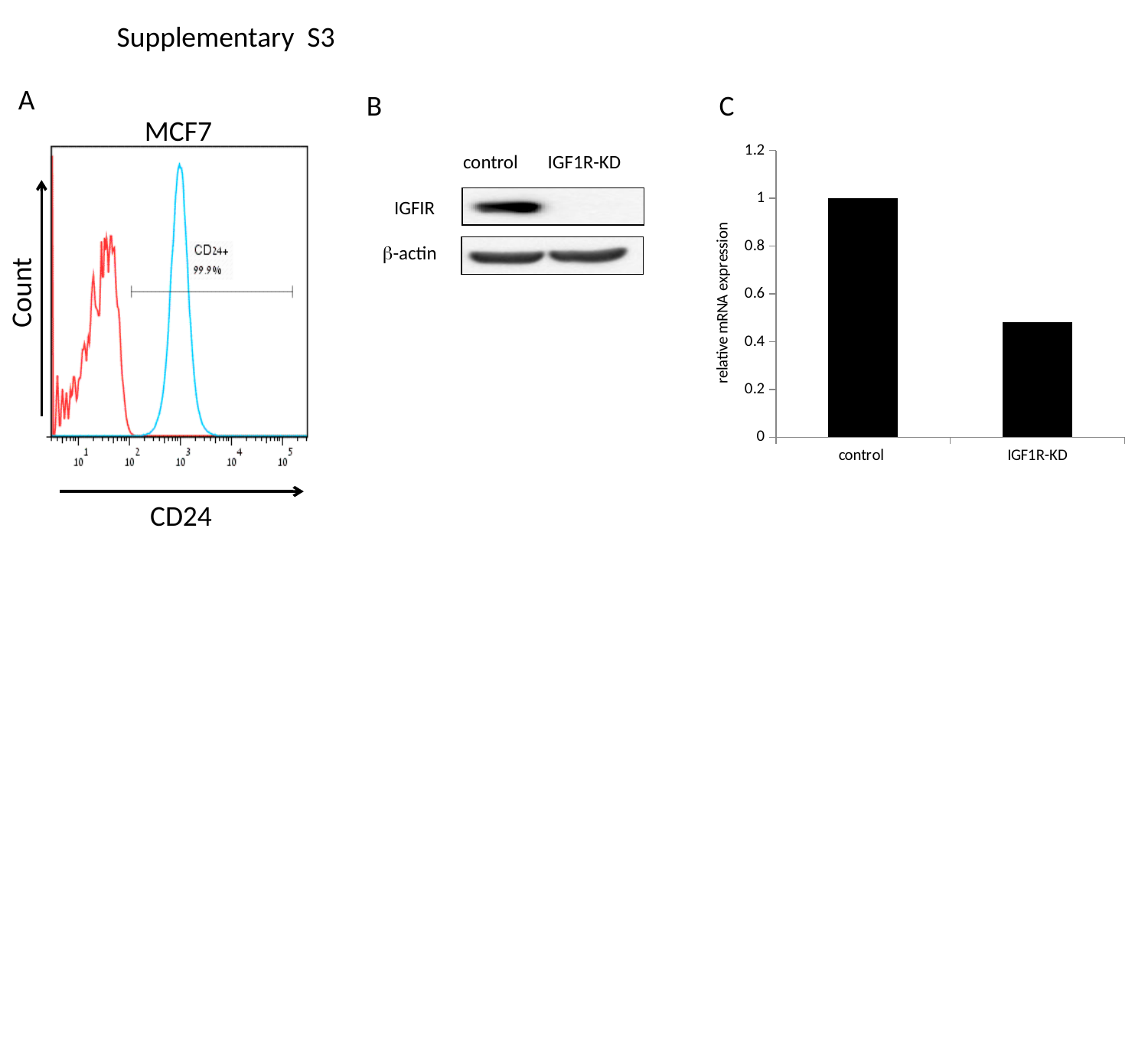

Supplementary S3
A
B
C
MCF7
### Chart
| Category | |
|---|---|
| control | 1.0 |
| IGF1R-KD | 0.48 |
control
IGF1R-KD
IGFIR
b-actin
Count
CD24
